# Exploration of patients and multidisciplinary point of view about the self-care needs of patients with a stoma: a qualitative study

**DOI:** 10.1186/s12885-026-15920-8

**Published:** 2026-04-11

**Authors:** Chandra Isabella Hostanida Purba, Rina Anggraeni, Tuti Pahria

**Affiliations:** 1 Universitas Padjadjaran, Bandung, Indonesia; 2 Hasan Sadikin Hospital, Bandung, Indonesia

**Keywords:** Digital education, Good health and wellbeing, Reduced inequalities, Self-care, Self-efficacy, M-Health, Ostomy

## Abstract

**Background:**

Colorectal cancer patients with a stoma face various physical, psychological, and social challenges in self-care. Low self-efficacy, limited access to continuous education, and social stigma often hinder improvements in their quality of life. Identifying patients’ self-care needs is a crucial first step in designing digital-based interventions through the Stoma m-Health application.

**Objective:**

This study aims to explore the self-care needs of colorectal cancer patients with a stoma from the patient and healthcare worker perspectives as a basis for developing the stoma m-Health application.

**Methods:**

This study employed a qualitative approach using semi-structured interviews. Ten participants (six stoma patients and for healthcare providers) were selected through purposive sampling at Dr. Hasan Sadikin General Hospital, Bandung, Indonesia. Data were collected via interview and analysed using the Husserian phenomenological approach. Research was maintained through rigor, trustworthiness dan credibilityrigor.

**Results:**

Data analysis yielded seven main themes: (1) physical barriers to stoma care (pouch leakage, skin irritation) (2) limited self-care knowledge and skills (3), family support as a key factor (4), stigma and psychosocial barriers (5), the need for easy and continuous access to information (6), patient coping and adaptation strategies, and (7) the potential of digital technology as an educational media.

**Discussion:**

This qualitative study revealed that patients with cancer and stoma face complex barriers to self-care, yet they also have the potential to strengthen their self-efficacy through digital educational support. While these findings provide a crucial foundation for developing a stoma m-Health application to enhance self-efficacy, it is essential to consider potential barriers, such as technological literacy and access disparities. Addressing these factors during development will help ensure that the application is inclusive and effective.

**Conclusions:**

These findings strongly demonstrate that patients with cancer and a stoma require practical education, robust social support, and interactive, easily accessible digital media to achieve independence and confidence in managing their self-care.

## Introduction

Global cancer data from 2020 identify colorectal cancer as the third most common cancer worldwide and a leading cause of permanent stomas [1]. Stomas resulting from gastrointestinal surgery significantly alter patients’ routines, including wound care, stoma bag management, and social adaptation [2]. The rising prevalence of colorectal cancer in Indonesia has led to an increasing number of patients living with a stoma. These patients face complex physical, psychological, and social challenges in their daily life, emphasising the need for credible, practical, and accessible educational resources tailored to stoma care to support their adaptation to stoma self-care [3].

A previous study in Indonesia found that patients living with a stoma cope by reconciling with their condition, seeking social support, and making daily adjustments [4]. When patients lack adequate information and support, they may lose self-efficacy, experience stress, and face a higher risk of complications such as skin irritation and stoma pouch leakage [5]. Comprehensive interventions to improve self-efficacy among stoma patients remain limited [5]. Most Indonesian research focuses on medical or physical complications, while psychological and social issues are less explored. Patients also face stigma, embarrassment, low self-esteem, and isolation [4]. Some withdraw socially due to concerns about pouch leakage or odor [5], which adds to their emotional burden and makes adaptation more difficult [6].

Bandura’s theory, initially known as social learning theory and later expanded into social cognitive theory, explains that people learn through observation of others (models) and through internal cognitive processes rather than solely through direct experience or direct reinforcement [7]. Learning occurs through modelling and involves the following stages: attention, retention, reproduction, and motivation. Self-efficacy, or a person’s belief in their ability to succeed, strongly influences behavior. Watching instructional videos and following step-by-step demonstrations seems to increase the patient’s belief in their ability to do self-care. Positive coping strategies, such as adapting to the stoma, have been shown to accelerate patient adjustment [4]. In addition, preparing patients with online-based ostomy care during discharge planning can reduce post-surgical complications [8], as patients demonstrate improved ability to do self-care at home.

Multiple studies have reported self-care behavior is influenced by various factors including knowledge, motivation, intention, social support, socio-demographic status, and clinical conditions [7]. Most of these factors act as mediators or moderators by shaping self-efficacy, defined as the belief in one’s ability to carry out necessary actions, that will eventually affect behavior [9]. Bandura’s Social Cognitive Theory asserts that self-efficacy is a direct predictor of health behaviors, including self-care, because it determines an individual’s confidence to initiate, maintain, and overcome barriers to action [7]. Self-efficacy is an individual’s belief in their ability to organise and carry out the necessary actions in a given situation [7]. High self-efficacy leads to successful self-care, even in challenging chronic conditions, such as living with a stoma. Interventions that boost self-efficacy help improve adherence, quality of life, and independence among stoma patients [10]. Evidence from patients with chronic diseases indicates that adults with high self-efficacy are more likely to adhere to self-care practices in managing their illness [9].

Stoma patients often have low self-efficacy, which directly affects their ability to perform self-care [11]. One study found that individuals living with a stoma may experience identity disruption and loss of self-control, further reducing their post-surgery self-efficacy [11]. Patients require information about post-surgery changes, potential complications, and effective coping strategies [12]. Preoperative preparation, including psychological support, has been shown to lower the risk of postoperative complications and help patients adapt, cope with the stoma, and rehabilitate both physically and psychosocially [13].

Studies have shown that patients who do not receive continued education after hospital discharge often struggle to adjust to stoma care [11], which prolongs their dependence on caregivers. This highlights a knowledge gap on patients’ actual self-care needs. A recent study in Indonesia indicates that many patients living with a stoma rely heavily on family members for daily care due to fear, confusion, and low confidence [4]. Low self-efficacy is still a fundamental barrier to independence, affecting both quality of life and long-term self-care.

Social media increasingly influences how individuals make health decisions and improve care quality. For patients from remote areas with limited healthcare facilities, access to stoma care can be challenging. Telehealth nursing applications offer an opportunity to support self-care at home [14]. However, unreliable online resources may mislead patients and hinder proper care [6], which underline the importance of m-Health platforms recommended by professional healthcare providers. Telehealth can help patients monitor their health at home while reducing the burden on healthcare providers and family caregivers [8]. Understanding patients’ experiences and challenges is key to designing effective interventions [15]. An online program grounded in nursing practice can serve as a structured educational model [16]. Without adequate education, patients may feel insecure and unable to manage their stoma independently. Education during nursing process can improve the knowledge and skills of patient in ostomy care [17].

Little is known about the use of telehealth for stoma care in Indonesia, and most online stoma care resources are designed for non-Indonesian users. Preliminary studies are therefore essential to inform the development of digital technology-based interventions, such as a stoma m-Health application. This study explores the self-care needs of patients living with a stoma and examines the perspective of both patients and healthcare providers regarding the use of an m-Health application to support stoma self-care at home. The research question guiding this study is ‘What are the experiences of patients living with a stoma in performing self-care, and what type of stoma health application do patients and healthcare providers consider necessary to support self-care at home? Two of seventeen Sustainable Development Goals (SDGs) are Reduced inequalities and Good health and well-being [18]. This research will help to decrease inequalities and increase quality of life among patients with ostomy.

## Method

This paper reports only the qualitative stage of a mixed-method study consisting of three stages, conducted to inform the development of a stoma m-Health application [19]. In this stage, interviews were conducted to explore what type of care application should be developed for individuals living with a stoma to support their stoma self-care and strengthen their self-efficacy. The study examined patients’ experiences, abilities, and self-care challenges in performing stoma self-care. The subsequent stages, Stage 2 (a Design Thinking approach to develop the application based on findings from Stage 1) and Stage 3 (a quantitative study to test the effect of the application on the self-efficacy of patients living with stoma in doing their self-care before and after using the application) will be reported separately. The Husserlian phenomenological approach was used in analyse the data [20] because it enables researchers to uncover the real needs of patients adapting to a stoma while minimising bias. This approach allows researchers to understand the subjective meanings embedded in the experiences of patients living with a stoma, which is vital for designing an effective intervention application [21]. Presenting phenomenological findings can also influence readers’ understanding of the emotional reality faced by the patients [22]. The findings from this stage are expected to to reflect patients’ need and expectation for stoma self-care support through the use of a digital tool, which will inform and guide the development of such tool. The interviews helped identify barriers, coping strategies, and patient expectations for care [23], providing evidence to support the development of m-Health applications [24] that align with Indonesian cultural contexts and patient needs [25].

The research team determined the number of participants based on the principle of saturation, which occurs when repeated data collection yields no significant new information [26]. Saturation was considered reached when no new code emerged from participants in both patients and healthcare provider groups. Recruitment ceased when once the researchers consistently received similar information from participants, and their responses reflected identical meanings regarding their needs and experiences related to stoma care.

Six patients living with stoma and four healthcare providers involved in stoma care participated in this study. Data collection concluded once all interviews were completed. The audio recordings were transcribed verbatim, then analysed using NVivo qualitative data analysis software version 11.0 to develop themes and subthemes. The primary instrument for this study was the researcher herself [27]. Recruitment ceased when participants consistently provided similar information, and no new meanings emerged regarding their needs and experiences related to stoma care.

Participants were selected using purposive sampling. Ten participants were recruited for semi-structured interviews: six patients living with a stoma and four healthcare provider group involved in stoma care. The inclusion criteria for patients were being 18 years or older and having lived with a stoma for at least three [3] months. Patients with terminal illnesses or cognitive impairment were excluded. Healthcare providers were eligible if they have at least 12 months of experience caring for patients with a stoma. Each participant was assigned a code indicating participant number, role, and gender: P = participant number, p = patient, F/M = female/male, and professional abbreviation for healthcare providers (e.g., Psy for Psychologist, Nur for nurse, etc.). For example, P1pF referred to Participant 1, a female patient, and P7Msurgeon referred to Participant 7, a male surgeon.

Husserlian Phenomenology was applied to construct themes from the collected data. Husserl’s method involves four fundamental processes in data analysis: intentionality, bracketing (Epoche), phenomenological reduction, and essence (objective) [28]. *Intentionalit*y refers to the idea that consciousness is always directed toward something; individuals are always aware of an object or experience. *Bracketing* involves suspending judgments, assumptions, and prior knowledge to view the phenomenon as it is, rather than through preconceived interpretations. Bracketing begins during data collection and continues throughout analysis, allowing researchers to identify the essential meanings of participants’ experiences [29]. Researchers engaged in personal reflection to recognise and set aside preconceptions before beginning analysis. *Phenomenological reduction* then focuses on isolating the natural attitude to reach a clearer awareness of the essence of the experience. This reduction occurs throughout the research process, including fieldwork and report writing. During this stage, researchers gathered interview data, documented field notes, and organized the material by separating and classifying it into relevant topics. The reduction process involved summarising, coding, and identifying themes that captured the core of the phenomenon. The *objective* of this approach was to discover the basic structure of human experience (descriptive), rather than interpreting or explaining it scientifically.

Themes were constructed directly from the raw data using an inductive approach. Verbatim transcripts were read line by line to identify the codes, which were generated from participants’ own words using the NVivo software. The researcher read all transcripts multiple times to gain familiarity with the data and to obtain a general sense of the participants’ experiences. Codes with similar meanings were grouped together, then organised into potential sub-subthemes, and, subsequently, into subthemes. Each sub-sub-subtheme was supported by statements from at least two participants. The researcher extracted all statements relevant to the phenomenon under study directly, focusing on participants’ perceptions, feelings, and experiences. Subthemes were then clustered to form initial themes that reflected broader patterns across the data. Significant statements were interpreted to generate “formulated meanings,” capturing both explicit and implicit meanings embedded in the original statements. These formulated meanings were further grouped into larger thematic clusters representing conceptual patterns or commonalities across participants’ experiences. Themes were refined through reviewing the initial themes to ensure that the themes were well defined and answered the research question. The researcher then synthesised the themes into a comprehensive, rich, and exhaustive description of the phenomenon under study, incorporating all aspects of participants’ experiences, identifying the essential or “fundamental structure” of the experience, which captures the core meanings that define the phenomenon. This was followed by further reviewing and refining to be able to obtain the final themes.

Bias was managed through reflexive field notes. The researcher documented the entire interview process, including participants’ non-verbal expressions, and clarified meanings with participants. Reflexivity supports rigor and trustworthiness [28]. The researcher kept a reflexive diary throughout data collection and analysis, noting any mistakes during the interview, wrote down the verbatim line-by-line to see the codes and nodes, and then making decisions on final themes. A table linking research questions and participants’ responses were used to maintain consistency. The second author generated the initial codes and sub-themes. while the first author developed subthemes, themes, and initial themes. All authors discussed the coding and thematic structure until consensus was reached. Any disagreements were resolved through collaborative discussion.

Seeking Verification (Member Checking) was conducted by returning the final themes to the participants to verify whether the findings had accurately and authentically reflected their original experiences. This step ensures the rigor of the analysis. The researcher presented the themes directly to the participants and asked whether their thoughts had been represented in them. All participants confirmed that the themes and subthemes had captured their thoughts, thus no change was required. Feedback were collected on the same day. Three patients and four healthcare providers reviewed the themes and agreed with the final themes.

### Ethical considerations

In accordance with the Declaration of Helsinki [30], the researcher upheld the key ethical principles: participant protection, informed consent, independent ethical review, qualified supervision, and minimisation of risk. Respect for persons, beneficence, and justice were ensured by considering the vulnerability of the community involved. Privacy and confidentiality are maintained through informed consent and the anonymisation of transcripts and reports. Participation in the study was voluntary, and participants could withdraw at any time without consequences. Ethical approval was obtained from the Research Ethics Committee of Universitas Padjadjaran (Number:1259/UN6.KEP/EC/2025). All respondents received an information sheet on the study and signed a written informed consent before the study began.

## Results

Data were collected from ten respondents who participated in semi-structured interviews. Most respondents were aged 45–59 (60%), female (80%), had a low to secondary education level (50%), were unemployed (50%), and had 25–60 months of experience with stoma care (50%). Six respondents were patients living with stomach, and four were healthcare providers (a physician, two nurses, and a nutritionist). Seven prominent themes were identified, illustrating both the potential and challenges of patient self-care. Each subtheme was supported by statements from at least two participants. The themes were organised into subthemes and subcategories, as presented in Table 1 content analysis.

**Table 1:** Content analysis themes and subthemes

No	Theme	Subtheme	Code	Verbatim	Participant
1	Physical barriers to stoma care	Pouch leakage	Difficulty maintaining dryness	“My pouch often leaks, sometimes leaking through my clothes while I sleep.”	(P3pF)
			Inadequate equipment	“The equipment isn’t quite right. “The bag adhesive sometimes does not stick for long, so it comes off quickly.”	(P6pM) (P4pF)
		Skin irritation	Wounds around the stoma	“The skin around the stoma is often red and sore. I’m afraid it will get worse.”	(P3pF)
			Pain during wound care	“I feel pain during wound care, so I skip it.”	(P2pF)
				“During treatment, and especially upon discharge, patients sometimes need nutrition to repair their wounds, so they can heal quickly and recover healthily. For example, nutrition is needed for nutritional care at home.”	P10FNutritionist
2	Limited self-care knowledge and skills,	Limited knowledge	Do not know how to do proper care.	“At first, I was confused. I didn’t know how to clean my stoma properly.”	(P4pF)
				“Usually, the choice of food containing protein is mainly because what we patients have education and prohibitions for example, the same as prohibitions at home, for example, belief, the point is the belief in reducing animal products such as eggs because they can cause itching or animal products can make patients itchy, even though actually high protein is needed for wound healing, it’s just a wrong perception among patients, right?”	P10FNutritionist
				Important information to convey before surgery is that there are several things we as nurses must prepare. These include how to prepare the patient preoperatively or perioperatively for a stoma, how to prepare the stoma pouch for use intraoperatively, and, of course, how to prepare the patient for the postoperative or rehabilitation phase.	(P9FNurs)
			Minimal information about complications	“If my skin itches or oozes, I don’t know what to do.	(P5pF)
				“Telekonsul is very important after home, spesially for patient after colostomy’.	P9FNur
				“That’s representative enough to be used as content for the app. Because, you know, it’s healing, and I forgot. Usually, stoma patients are restricted at home, but it’s not allowed to do these things, even though it’s absolutely necessary to do it in the hospital.”	P9FNurs
				Teleconsultation can help provide this information directly, which may not have been conveyed or forgotten before or during patient care. Furthermore, teleconsultation also aims to monitor patient conditions. With teleconsultation, medical personnel can monitor patients’ conditions more regularly, checking for signs of infection or complications, for signs of inflammation or other complications. This telecommunication is very helpful in ensuring that patients do not experience more serious health problems after returning home. Furthermore, teleconsultation also provides a sense of security and comfort, thereby increasing patient confidence.	(P7MSurgeon)
		Limited skills	Technical error	“I once put the bag on wrong, and it leaked again.”	(P1pF)
			Lack of independent practice	“I rarely do it by myself, usually with the help of a nurse or family member.”	(P2pF)
				“The most important information that must be provided to patients after stoma surgery is, of course, how to care for their stoma independently. “Continuous education is needed before, during, and after surgery, especially regarding home care.”	P9FNurs
3	Family support as a key factor	Practical help	The role of spouse/child Parental support	“My wife always helps prepare the tools and change the bags.”	(P5pF)
				“When I’m tired, my parents will help me clean.”	(P1pF)
				“I rarely practice alone, usually with the help of a nurse or family member.”	(P3pF)
		Emotional support	Family motivation	“My children encourage me to stay strong.”	(P3pF)
		Spiritual support	Prayer & religious encouragement	“The family always reminds us to be patient and pray a lot.”	(P1pF)
4	Stigma and psychosocial barriers,	Feelings of shame	Feelings of inferiority	“I rarely leave the house; afraid people will find out I have a stoma.”	(P6pM)
			Loss of self-confidence	“I don’t feel normal anymore.”	(P3pF)
				“The stoma application is certainly very useful for patients who have undergone stoma surgery because patients get faster and easier information about how they will care for their stoma, what they need to pay attention to in their stoma and also what complications can occur in the stoma that has been operated on, which has been provided, which has been received by the patient.	P7MSurgeon
				“From the psychologist’s perspective, the patient’s unmet needs are usually those of a consultation, perhaps at the beginning of the education session, many people didn’t understand, or when the education was given, they weren’t prepared, so the material presented didn’t reach the intended audience. They didn’t understand it, so when they were asked again, they didn’t.”	P8FPsy
				However, the detailed needs of pre-operative patients have not been optimally met. Pre-operative counseling should encompass cognitive, affective, and psychomotor aspects. Currently, this is not being implemented optimally.	P7MSurgeon
		Social barriers	Limited interaction	“Don’t dare to hang out with friends because I’m afraid it will leak.”	(P4pF)
				“Knowing ways to deal with stress related to the process of accepting changes due to a stoma	(P8FPsy)
		Discrimination		“Sometimes people are disgusted when they find out I have a stoma.”	(P4pF)
5	The need for easy and continuous access to information,	Medical information	Care guidelines	“If I can have a guidelines book or other media.”	(P1pF)
				“Yes, we were given information, so that it would run smoothly and reduce the pain. Rather than keeping it low, it’s better to keep it high so that you don’t have to endure the pain continuously, so it’s better to use a stoma.”	(P3pF)
				We provide information in general terms, but the goal is to convey the purpose and indications for stoma creation. What are its characteristics and what is done to the patient if a stoma is created? When we urinate or defecate, we do so through the back door. Because the back door is problematic, it is temporarily unusable or needs to be repaired. Therefore, an alternative exit route is needed, through the abdominal door.	P7MSurgeon
				Then, besides that, the type of surgery that will be performed on the patient, namely the type of perioperative stoma, which if we classify whether the stoma is made as a colostomy, ileostomy, or urostomy. Because later the type of stoma surgery will affect the preparation for post-operative care.	P7MSurgeon
				The second is to recognize post-stoma creation complications such as ischemic stoma, necrotic stoma or parastomal hernia and stenosis.”	P7MSurgeon
		Ongoing education		“Education stopped after discharge, so I was confused at home.”	(P1pF)
				“Teleconsultation is important for patients who have undergone stoma surgery, especially if the patient lives quite far from the hospital. For some problems, such as general stoma care, a stoma specialist nurse may suffice. However, if complications arise after stoma creation, a digestive culture consultant may be needed to provide telecommunication services. Some patient complaints.”	P7MSurgeon
				“The stoma application is certainly very useful for patients who have undergone stoma surgery because patients get faster and easier information about how they will care for their stoma, what they need to pay attention to in their stoma and also what complications can occur in the stoma that has been operated on, which has been provided, which has been received by the patient.	P9FNurs
				“YES, it can depend on which segment we are targeting. If it’s under 50 years old, it might be suitable. Those with junior high or high school education might be able to.”	P10FNutritionist
		Easy access	Online media	“Searching online has confused me; the information is mixed.”	(P2pF)
				“In my opinion, due to the limited number of ETNs in a hospital unit, or the ability to provide good education, and the limited time available for nurses, providing information on patient self-care using the mobile heart smartphone app is essential. In today’s world, with so many limitations, busy schedules, and busy routines, we need easy access to information anytime, anywhere. Therefore, in my opinion, providing information on self-care using the mobile hub smartphone app is essential.”	P9FNurs
6	Patient coping and adaptation strategies	Emotional coping.	Self-acceptance.	“I’ve learned to accept the situation, even though it’s difficult.”	(P1pF)
				“Content that motivates patients to actively participate in their care process. Furthermore, creating relevant content for patients, especially those new to stoma care, is highly beneficial. This content not only meets patients’ needs for information and emotional support, but also has a positive impact, improving their appearance, confidence, and quality of life. With easily accessible information, patients can better care for their stomas independently and be better prepared to face the challenges ahead. Therefore, content related to stoma care creators has a significant impact on patient needs.”	P8FPsy
		Independent practice	Gratitude & patience	“If you are impatient, it just adds to your stress.” “Now I am eating regularly, so my stoma doesn’t fill up quickly.”	(P3pF)
				Practicing skills and understanding the care process can reduce anxiety and fear. Of course, as nurses, preparing patients to be independent is not easy.	P9FNurs
		Behavioral coping	Activity modification	“I try to change things up a little bit on my own, so I get used to it.”	(P6pM)
			Self-training:	“I’ll try changing it a little bit myself, just to get used to it.”	(P2pF)
				“Knowledge impacts self-confidence, so improving knowledge first will only increase self-confidence.”	P9FNurs
				“Some of the patient complaints during treatment at RSHS, both in the ward and in the polyclinic, seem to be enough as a basis for creating the stomaku application. To further deepen what needs to be included in the application, patients usually have to be followed up both while in the ward and after returning home from treatment so that they can get more information about what problems might occur in patients who have undergone stoma surgery.”	P7MSurgeon
				“Food selection, perhaps knowledge, and then economic level, but economics can also influence food selection. That’s the most important thing: knowledge and economics.”	P10FNutritionist
7	Potency of digital technology as education’s media	Digital media	Education Practical guide:	“If there are guidelines step by step, it must be easy.”	(P5pF)
			Video tutorial	“It would be clearer if there were a video on how to clean the stoma.”	(P4pF)
				“It’s very necessary because nowadays, everyone’s gadget media is inseparable from gadgets, especially since it’s the gadget season, which means it’s more necessary than paper because people just look at paper and say, ‘What’s this?’ Throw it away, throw it away. If it’s a gadget, it can be opened and read at any time.”	P9FNurs
		Q&A feature	Interactive support	“If possible, there is a nurse that I can contact via the application.”	(P4pF)
			Anytime access	“I want an app that I can access anytime, from home.”	(P2pF)

### Themes and subthemes

#### Theme 1. Physical barriers to stoma care

This theme was constructed from two sub-themes: pouch leakage and skin irritation. Pouch leakage was described as a difficult-to-manage problem due to inadequate equipment.

*“My pouch often leaks*,* sometimes leaking through my clothes while I sleep.”(P3pF)*.

*“The equipment isn’t quite right. “The bag adhesive sometimes does not stick for long*,* so it comes off quickly.”(P7MSurgeon)*.

Skin irritation was reported as a consequence of the stoma bag, causing participants to experience pain during wound care.

*Wounds around the stoma added pain for the patients.” P9FNurs*.

*“The skin around the stoma is often red and sore. I’m afraid it will get worse.” (P9FNurs)*.

*“I feel pain during wound care*,* so I skip it*.”(P2pF).

#### Theme 1. Limited self-care knowledge and skills, consisting of two subthemes and four sub-subthemes respectively

Limited knowledge was reflected in participants’ lack of understanding on how to perform proper stoma care and their minimal awareness of potential complications. Limited skills, on the other hand, were associated with technical errors and a lack of independent practice.

*“At first*,* I was confused. I didn’t know how to clean my stoma properly.”(P4pF)*.

*“If my skin itches or oozes*,* I don’t know what to do”(P6pM).*

*“I once put the bag on wrong*,* and it leaked again.”(P1pF)*.

“*I rarely do it by myself*,* usually with the help of a nurse or family member*.”(P2pF).

#### Theme 3. Family support is a key factor

Family support was defined as the practical assistance provided by the spouse or children. Family also offered emotional support through encouragement and motivation. Spiritual support was described arising from prayer and religious reinforcement.

*“My wife always helps prepare the tools and change the bags.”(P5pF)*.

*“When I’m tired*,* my parents will help me clean.”(P10FNutritionist)*.

*“I rarely practice alone*,* usually with the help of a nurse or family member.”(P3pF)*.

*“My children encourage me to stay strong.”(P8FPsy)*.

*“The family always* reminds us to be patient and pray a lot.”(P1pF).

#### Theme 4 stigma and psychosocial barriers

Feelings of shame were expressed as a sense of inferiority and a loss of self-confidence. Participants reported feeling too embarrassed to disclose their condition to others and, in some cases, even avoided going out. Subthemes: Social barriers were also described, as participants limited their interactions because they perceived that others were avoiding them. One participant even perceived discriminatory behavior from others.

*“I rarely leave the house; afraid people will find out I have a stoma.”(P6pM)*.

*“I don’t feel normal anymore.”(P3pF)*.

*“Don’t dare to hang out with friends because I’m afraid it will leak.”(P7MSurgeon)*.

*“Sometimes people* are disgusted when they find out I have a stoma.”(P4pF).

#### Theme 5. Need for easy and continuous access to information

Participants expressed their need for easy access to continuous information and a continuum of care. They expected to be able to access easily accessible medical information, care guidelines, ongoing education, and online media.

*“If I can have a guidelines book or other media.”(P8FPsy)*.

*“Education stopped after discharge*,* so I was confused at home.”(P1pF)*.

*“Searching online has made me confused; the information is mixed*.”(P2pF).

#### Theme 6. Patient coping and adaptation strategies

This theme was represented through emotional coping, independent practice, and behavioral coping. The strategies used for applying these coping mechanisms included self-training, self-acceptance, gratitude and patience, and activity modification.

*“I’ve learned to accept the situation*,* even though it’s difficult.”(P9FNurs)*.

*“If you are impatient*,* it just adds to your stress.”(P6pM)*.

*“Now I am eating regularly*,* so my stoma doesn’t fill up quickly.”(P8FPsy)*.

*“I try to change things up a little bit on my own*,* so I get used to it.”(P7MSurgeon)*.

*“I’ll try changing it a little bit myself*,* just to get used to it*.”(P2pF).

#### Theme 7. Potential of digital technology as education’s media

Participants expressed their expectations for technology to function as an educational medium and a practical guide, including features such as digital media, video tutorials, Q&A functions, interactive support, and anytime access.

“*If there are guidelines step by step*,* it must be easy.”(P5pF)*.

*“It would be clearer if there were a video on how to clean the stoma.”(P9FNurs)*.

*“If possible*,* there is a nurse that I can contact via the application.”(P8FPsy)*.

*“I want an app that I can access anytime*,* from home*.”(P2pF).

Assessing the statistical relationship between outcomes and participants’ population’s socio-demographic background was not applicable because the data were qualitative. Patient participants provided more detailed account of their lived experiences as stoma users, while healthcare providers offered insights into what they thought the patients need during hospital care.

Using Husserlian phenomenological analysis, four stages of meaning emerged from participants’ accounts.

First, *intentionality* was reflected in participants’ awareness of their need for digital tools to support stoma self‑care at home. Both patients and healthcare providers recognized that self‑care requires accessible guidance beyond the hospital setting.

Second, *bracketing (epoché)* revealed the core experiences of living with and caring for a stoma at home. Patients described the challenges, uncertainties, and emotional burden of performing self‑care, while healthcare providers highlighted the gaps they observed in patients’ knowledge and the types of digital support patients would benefit from.

Third, phenomenological reduction uncovered significant statements that illustrated the difficulties of home‑based stoma care. Patients described discomfort, fear of leakage, and limited confidence in managing their stoma independently. Healthcare providers frequently reported encountering infected or leaking stomas during follow‑up visits. Across both groups, participants expressed a clear desire for online guidelines and digital tools to support daily self‑care. These significant statements were clustered into themes that reflected the essential elements of their experiences.

Finally, the objective stage captured participants’ understanding of the meaning and lifelong responsibility of stoma self‑care. They recognized that consistent care is necessary to prevent complications and maintain social confidence, particularly in avoiding leakage or odor. Participants believed that digital tools would help them perform daily self‑care more effectively and reduce embarrassment in social situations. Through the synthesis of meanings and essences, participants envisioned using M‑health guidance to support consistent, long‑term stoma care at home.

## Discussion

The results indicate that stoma patients face significant physical and psychological challenges, have limited knowledge, and are highly dependent on family support. They also encounter various barriers to self-care, encompassing physical, psychological, social, and informational constraints. Seven key themes emerged: physical barriers, limited knowledge, family support, psychosocial stigma, need for access to information, coping strategies, and potential use of digital technology as an educational tool. These findings align with a previous study demonstrating the tendency of stoma patients to struggle with practical adjustments in daily care and depend on family members for support [4].

Despite the challenges they face, patients expressed high hopes for m-Health-based digital educational interventions that could enhance their self-efficacy and independence in stoma care. This present study also demonstrates that patients need easy, practical, and ongoing access to information. The use of positive coping strategies, such as self-acceptance and spiritual support, has been shown to facilitate patients’ adaptation process. Furthermore, most respondents welcomed the idea of ​​using digital technology as an educational medium, viewing it as a way to bridge the information gap and provide more applicable care guidance. Thus, this study provides a strong foundation for the development of m-Health-based educational interventions that can improve the self-efficacy of patients living with a stoma in performing their self-care practices.

The psychological burden of cancer and survivorship is also reported among patients. Cancer patients frequently experience psychological distress, adversely affecting their treatment adherence and outcomes. This chronic psychological stress and oncogenesis highlight the complex multisystem interactions and underscores the need for multimodal interventions such as psycho-behavioral interventions, pharmacotherapy, socio-environmental improvements, and other complementary strategies [31].

Family caregivers also reported burdens associated with their caregiving roles. Their involvement in stoma care is shaped by broader socio-cultural expectations surrounding family responsibility in long-term care. Recent evidence from China shows that family structure influences long-term care arrangements in the context of aging and chronic illness. In rural areas of China, parents whose first child is a boy are more likely to receive care at home rather than in a nursing home [32]. It is rooted in Asian traditional wisdom that the son has the responsibility to take care of their parents. Most caregivers in Asian are family members. However, unpaid family caregivers frequently report high levels of burdens across multiple domains as a result of their role [33]. Family caregivers sacrifice their personal lives, time, and money to take care of the family member, which places them at high risk of depression placed them in high risk of depression [34]. Hence, the family caregivers also need adequate supports. Physical barriers, such as stoma pouch leakage and skin irritation, were among the most frequently reported problems by participants. These conditions not only cause physical discomfort but can also trigger anxiety and reduce patients’ quality of life. Previous studies have confirmed that peristomal skin complications significantly affect patients’ confidence in self-care [34]. These findings highlight the importance of education and practical skills in enabling patients to overcome these physical barriers effectively.

Limited knowledge and skills were also significant challenges in this study. Many patients felt they did not receive ongoing education after hospital discharge. These findings align with previous research showing that stoma education often ends during inpatient care, making it difficult for patients to continue independent self‑care at home [35]. These findings highlight the gap between educational needs and the educational services available at the healthcare facilities. Digital technology, including online platforms, is anticipated to reduce unnecessary travel and facilitate easier access to care for patients in diverse locations [36]. Issues that may be important for young patients include future care, autonomy, as well as informational and emotional needs. Facilitating death-education programs for advanced cancer patients can help to improve the quality of life [37].

Family support emerged as a key factor assisting patients in self-care [33]. Spouses, children, and other family members have been shown to provide both emotional motivation and practical assistance. This finding is consistent with those of Rajah et al. (2021), who suggested that social support, particularly from family, plays a crucial role in enhancing the self-efficacy of patients living with a stoma [38].

Emotional support has also been shown to reduce stress, anxiety and depression, thereby improving patients’ quality of life [33]. Stigma and psychosocial barriers also emerged in patient narratives. Some respondents felt inferior, embarrassed, and socially withdrawn due to their stoma. This finding aligns with international research indicating that social stigma can worsen patients’ psychological well-being and reduce motivation for self-care [39]. These findings highlight the need for interventions that focus not only on physical aspects but also on psychosocial support. Advance care planning should also integrate communication about the future care and autonomy [40].

The need for easy and continuous access to information emerged as another important theme. Respondents expressed that the information available online is often confusing due to inconsistency. This result aligns with existing literature that revealed the need for credible, practical, and accessible educational resources and personalized support for patients living with a stoma [41].

Social media is increasingly used to help users make decisions about health issues and improve the quality of care. The limited healthcare facilities and the patient’s remote location prevented the patient from accessing stoma care. Limited healthcare facilities and remote geographic locations often prevent patients from accessing specialized stoma care. Telehealth nursing applications have been shown to be valuable tools for supporting home‑based stoma self‑care [14], although the internet al.so poses risk when information sources are untrustworthy or unreliable [6].

Digital technology-based education offers a promising solution to bridge this information gap [40]. Digital health tools are considered both acceptable and scalable. Previous studies have identified factors that facilitate patient adoption of online medical advice, particularly within team-based online consultation models [42]. Patients tend to trust multidisciplinary teams, which increases their confidence in following the recommendations provided by the team. Remote monitoring has also shown broader relevance in chronic device‑related conditions; for example, peritoneal dialysis patients have benefited from remote antibiotic concentration monitoring, which successfully resolved infections. Other online self‑monitoring interventions have demonstrated efficacy rates as high as 88.9% compared with non‑monitored groups [43].

Patients’ coping and adaptation strategies varied, ranging from prayer and self-acceptance to reliance on family support. This finding is consistent with Bandura’s (1997) theory of self-efficacy, where a person’s confidence in overcoming challenges plays a crucial role in successful self-care [7]. Patients responded positively to the potential use of digital technology as an educational tool, anticipating applications that offer practical guidance, educational videos, and opportunities for interaction with healthcare professionals. This result aligns with previous research among patients with type 2 diabetes, showing that m-Health-based self-management is effective in improving patients’ self-efficacy, with patients demonstrating their ability to self-management at home [44]. Participants were willing to accept M-Health if it met their needs and were supported by their environment [45].

Therefore, the development of digital applications has significant potential to drive innovation in modern nursing services [46]. In addition, telehealth counselling provides a continuum of psychological care for the patient [47]. Research and Development for this technology should begin by understanding the public’s expectations for the tools they use daily [48]. Technology development must also adhere to research ethics to ensure that the outcomes can be applied in practical life [49]. Innovation is likewise needed in the design, conduct, and reporting of patient cases in nursing care [50].

Previous research has demonstrated that providing individuals with the opportunity to express their concerns and receive responses, such as through chatbots, and enhances their access to public services [51]. This finding suggests that if patients have the opportunity to express their concerns and be heard through e-health, they will have easier access to healthcare services [52]. The primary finding of this study is that patients continue to experience significant challenges in addressing technical issues, such as stoma pouch leakage and skin irritation. Furthermore, a lack of ongoing education leads patients to be less confident in managing their own stoma care. Family support proved to be a key factor in maintaining continuity of care, while social stigma exacerbated patients’ psychological well-being.

This study highlights the crucial role of healthcare professionals in providing consistent support, both during hospitalization and after discharge. Broader structural and geographic determinants also influence access to care. Indonesia, as an archipelago with around 13,000 islands [52], faces challenges in distributing health equipment and medical devices. Health-system and regional inequities in access to specialized stoma care underscore the need for regional health policy development.

### Strength and limitations

The limitation of this study is that it was conducted in a single hospital, so the findings cannot be generalized to a broader population. However, this study makes an important contribution by identifying the needs, barriers, and expectations of patients living with a stoma and offers a foundation for developing more effective digital-based educational interventions. Therefore, the results of this study not only enrich the understanding of the experiences of patients living with a stoma but also make practical contributions to the development of m-Health tools and patient-centered nursing services.

## Conclusions and recommendations

Self-care for stoma patients requires a comprehensive approach that encompasses not only medical but also psychological and social aspects. Seven key themes emerged: physical barriers, limited knowledge, family support, psychosocial stigma, the need for access to information, coping strategies, and the potential use of digital technology. Based on these findings, the development of digital-based educational programs is recommended, particularly those that offer anytime access, involve families in the educational process, and provide psychosocial support services to address stigma and anxiety. Further research with a larger and more diverse sample is needed to enhance the generalizability of these findings.

## Data Availability

The datasets used and/or analysed during the current study are available from the corresponding author on reasonable request.

## Abbreviations

e-health: Electronic health
m-Health: Mobile health applications
SDGs: Sustainable Development Goals
