# Correction to: Exploration of patients and multidisciplinary point of view about the self-care needs of patients with a stoma: a qualitative study

**DOI:** 10.1186/s12885-026-16785-7

**Published:** 2026-09-03

**Authors:** Chandra Isabella Hostanida Purba, Rina Anggraeni, Tuti Pahria

**Affiliations:** 1 Universitas Padjadjaran, Bandung, Indonesia; 2 Hasan Sadikin Hospital, Bandung, Indonesia

**Correction to: BMC Cancer 26, 647 (2026)**

**https://doi.org/10.1186/s12885-026-15920-8**

Following publication of the original article [1], the author reported a typo-error in the body text that needs to be rephrase, and the correct reference list:

1. Incorrect:
  In rural areas, parents with only one son are more likely to remain at home rather than enter a nursing home [31].
  Correct:
  In rural areas of China, parents whose first child is a boy are more likely to receive care at home rather than in a nursing home [32].
2. Incorrect:
  Integrating digital technology into advanced cancer care planning for young patients at home is encouraged [35] given their internet-based lifestyle.
  Correct:
  Digital technology, including online platforms, is anticipated to reduce unnecessary travel and facilitate easier access to care for patients in diverse locations [36].
3. Incorrect:
  Death education programs for advanced cancer patients may also be strengthened by incorporating psychosocial components [36], with attention to cultural background and patient-specific conditions [36].
  Correct:
  Facilitating death-education programs for advanced cancer patients can help to improve the quality of life [37].
4. Incorrect:
  Digital health tools are acceptable and scalable, and. previous studies have demonstrated factors that facilitate patient adoption of online medical advice, especially team-based online consultation models [42].
  Correct:
  Digital health tools are considered both acceptable and scalable. Previous studies have identified factors that facilitate patient adoption of online medical advice, particularly within team-based online consultation models [42].
5. Incorrect:
  This result aligns with previous research showing that m-Health-based education is effective in improving patients’ knowledge, attitudes, and self-confidence, as well as enabling self-management [44], with patients demonstrating their ability to self-manage at home.
  Correct:
  This result aligns with previous research among patients with type 2 diabetes, showing that m-Health-based self-management is effective in improving patients’ self-efficacy, with patients demonstrating their ability to self-management at home [44].
6. Delete the sentence and the reference.
  Health-system and regional inequities in access to specialized stoma care underscore the need for regional health policy development [53].
  Delete reference 53 from the reference list : Hu F, Yang H, Wei S, Hu H, Chen Y, Zhou H. Spatial networks of China’s specialized, refined, distinctive, and innovative medical device firms based on parent-subsidiary contacts: implications for regional health policy. Front Public Health. 2025;13:1676189
7. Correct reference list

1. Sung H, Ferlay J, Siegel RL, Laversanne M, Soerjomataram I, Jemal A, Bray F. Global Cancer Statistics 2020: GLOBOCAN Estimates of Incidence and Mortality Worldwide for 36 Cancers in 185 Countries. CA: A Cancer Journal for Clinicians. 2021;71(3):209-49.

2. Malik T, Lee M, Harikrishnan A. The incidence of stoma related morbidity - a systematic review of randomised controlled trials. Ann R Coll Surg Engl. 2018;100(7):501-8.

3. Kalev G, Marquardt C, Schiedeck T. [Stoma-associated complications-Prevention strategy and treatment concepts]. Chirurg. 2022;93(4):415-26.

4. Afiyanti Y, Milanti A, Wahidi KR, Fitch M. Embracing My Stoma: Psychosocial Adjustment of Indonesian Colorectal Cancer Survivors Living With a Stoma. Cancer nursing. 2025;48(2):E121-E8.

5. Rolls N, Yssing C, Bøgelund M, Håkan-Bloch J, de Fries Jensen L. Utilities associated with stoma-related complications: peristomal skin complications and leakages. J Med Econ. 2022;25(1):1005-14.

6. Addo K, Antwi E. The Impact of Social Media Usage on Healthcare Delivery among Nurses. E-Health Telecommunication Systems and Networks. 2024;13(04):67-86.

7. Bandura A. Social cognitive theory of self-regulation. Organizational Behavior and Human Decision Processes. 1991;50(2):248-87.

8. Li M, Yu K, Zhang Y, Mao A, Dong L. Impact of Discharge Planning Combined with "Internet Home Ostomy Care Platform'' in Patients with Permanent Colostomy after Rectal Cancer Surgery. Ann Ital Chir. 2024;95(4):699-707.

9. Tan F, Oka P, Dambha-Miller H, Tan NC. The association between self-efficacy and self-care in essential hypertension: a systematic review. BMC Fam Pract. 2021;22(1):44.

10. Bozkul G, CS S, Nur Arslan H. Nursing interventions for the self-efficacy of ostomy patients: A systematic review. Journal of Tissue Viability. 2024;33(2):165-73.

11. Gaol LML, Saraswati LQ, Gultom GS, Anita M, Firmansyah Y, Setiawan FV, Wijaya BA. Empowering Stoma Care: Enhancing Knowledge, Confidence, and Support Through Collaborative Approaches Between Doctors and Parental Communities for Children with Stomas. Journal of multidisciplinary healthcare. 2025;18(Issue 1):4089-98.

12. Burns E. Stoma-related complications: A report from the Stoma-Const randomized controlled trial, Correa-Marinez et al. Colorectal Dis. 2021;23(5):1028.

13. Silva N, Santos M, Rosado S, Galvão C, Sonobe H. Psychological aspects of patients with intestinal stoma: integrative review. Rev Lat Am Enfermagem. 2017;25:e2950.

14. Grewal U, Terauchi S, Beg M. Telehealth and Palliative Care for Patients With Cancer: Implications of the COVID-19 Pandemic. JMIR Cancer. 2020;6(2):e20288.

15. Chiang L-C, Chen W-C, Dai Y-T, Ho Y-L. The effectiveness of telehealth care on caregiver burden, mastery of stress, and family function among family caregivers of heart failure patients: a quasi-experimental study. International journal of nursing studies. 2012;49(10):1230-42.

16. Notoatmodjo. Promosi kesehatan dan perilaku kesehatan. 2018.

17. Momeni P, Darvishpour A, Mansour-Ghanaei R, Kazemnezhad L. The Effects of Education Based on the Nursing Process on Ostomy Self-Care Knowledge and Performance of Elderly Patients with Surgical Stoma. Nurs Res Pract. 2023;2023:2800796.

18. United Nations DoEaSA. The Sustainable Development Goals Report 2025. The Sustainable Development Goals Report 2025. 2025 20 March 2026. [cited 2026 20 March]; 1. Available from: https://sdgs.un.org/goals.

19. Lloyd P. Quality and qualities of design studies, design research and design. Design studies. 2023;84:101161.

20. Creswell JW. Editorial: Mapping the Field of Mixed Methods Research. Journal of Mixed Methods Research. 2009;3(2):95-108.

21. Busetto L, Wick W, Gumbinger C. How to use and assess qualitative research methods. Neurological Research and Practice. 2020;2(1):14.

22. Al-Sheikh Hassan M. The use of Husserl's phenomenology in nursing research: A discussion paper. J Adv Nurs. 2023;79(8):3160-9.

23. Fletcher AJ. Applying critical realism in qualitative research: methodology meets method. International journal of social research methodology. 2017;20(2):181-94.

24. Pathak S, Sen PK, Jayaram J, Miller JM. “Like Poles Repel While Unlike Poles Attract”: Contextual Performance Effects of Supply Base R & D, Focal Firm R & D, and Commercialization. Decision sciences. 2019;50(5):985-1030.

25. George AS, George J, Jenkins J. A Literature Review: Potential Effects That Health Apps on Mobile Devices May Have on Patient Privacy and Confidentiality. . E-Health Telecommunication Systems and Networks 2024;13:21.

26. Mishra S, Dey AK. Understanding and Identifying ‘Themes’ in Qualitative Case Study Research. South Asian Journal of Business and Management Cases. 2022;11(3):187-92.

27. Patton M. Qualitative Research & Evaluation Methods Integrating Theory and Practice FOURTH EDITION ed. Saint Paul, Minnesota: SAGE Publications, Inc; 2015 November 2014.

28. Priest H. An approach to the phenomenological analysis of data. Nurse researcher. 2002;10:50-63.

29. Kim H, Jun M, Rhee S, Wreen M. Husserlian phenomenology in Korean nursing research: analysis, problems, and suggestions. . J Educ Eval Health Prof. 2020;17:10.

30. Association WM. WMA Declaration of Helsinki – Ethical Principles for Medical Research Involving Human Participants Helsinky: the 75th WMA General Assembly, Helsinki, Finland, October 2024; 19th October 2024 [Available from: https://www.wma.net/policies-post/wma-declaration-of-helsinki/.

31. Huang B, An H, Wu H, Qiu Y, Su Y, Chen L, et al. Chronic Psychological Stress in Oncogenesis: Multisystem Crosstalk and Multimodal Interventions. Research. 2025;8:0948.

32. Gao H, Li R, Shen J, Yang H. Children’s gender and parents’ long-term care arrangements: evidence from China. Applied Economics. 2025;57(13):1510-25.

33. Purba C, Johnston B, Kotronoulas G. An Exploration of Family Caregivers’ Health Care Needs When Caring for Patients With Cancer in the Resource-Challenged Context of West Java, Indonesia. Seminars in Oncology Nursing. 2022;39(3):151369.

34. Al-Husban RY, Abu Shosha G. The Lived Experience of Jordanian Persons With a Stoma: A Qualitative Study. Gastroenterology nursing. 2022;45(5):300-9.

35. Purba C, Johnston B, Kotronoulas G. Developing a nurse-led palliative care intervention for adults with cancer and their family caregivers in the resource-challenged context of Indonesia. [PhD ]. Glasgow: University of Glasgow; 2025.

36. Zhang S, Li J, Zhang Y, Hu X. Effects of advance care planning for patients with advanced cancer: A meta-analysis of randomized controlled studies. International Journal of Nursing Studies. 2025;168:105096.

37. Su Y, Zhang S, Duan L, Hu X. The Effectiveness of Death Education on Death Anxiety, Depression, and Quality of Life in Patients With Advanced Cancer: A Meta-Analysis of Randomised Controlled Trials. Journal of Nursing Scholarship. 2025;57(6):941-56.

38. Rajah H, Chan C, Kong Y-C, Wong L-P, Bustaman R, Ho G-F, et al. Insights on emotional distress following cancer, sources of support and the unmet needs in a setting with limited supportive care services for people living with cancer. Supportive Care in Cancer. 2021;29(10):5811-9.

39. Dönmez A, Alici N, Borman P. Lived Experiences for Supportive Care Needs of Women with Breast Cancer-Related Lymphedema: A Phenomenological Study. Clinical Nursing Research. 2021;30(6):799-808.

40. Zhang X, Zhou L, Wang S, Fan C, Huang D. Facilitating Patient Adoption of Online Medical Advice Through Team-Based Online Consultation. Journal of Theoretical and Applied Electronic Commerce Research. https://doi.org/10.3390/jtaer20030231. Journal of Theoretical and Applied Electronic Commerce Research. 2025;20(3):231.

41. van der Steen JT, Lemos Dekker N, Gijsberts M-JHE, Vermeulen LH, Mahler MM, The BA-M. Palliative care for people with dementia in the terminal phase: a mixed-methods qualitative study to inform service development. BMC Palliat Care. 2017;16(1):28.

42. Adeniyi A, Adeniyi O, Samuel S. Revolutionizing Healthcare: The Impact of Machine Learning and Artificial Intelligence. E-Health Telecommunication Systems and Networks. 2024;13(04):87-91.

43. Cheng L, Kotronoulas G. How effective are self-management interventions in promoting health-related quality of life in people after primary treatment for breast cancer? A critical evidence synthesis. European Journal of Oncology Nursing. 2020;47:101776.

44. Ahn J, Yang Y, Kim J, Pahn J, Jang Y. Effectiveness of mHealth-Based Self-Management Interventions on Self-Efficacy in Patients With Type 2 Diabetes: A Systematic Review and Meta-Analysis. The Science of Diabetes Self-Management and Care. 2025;51(5):517-31.

45. Wester KL, Wachter Morris CA. Making research relevant: applied research designs for the mental health practitioner. Second ed. New York, NY: Routledge; 2025.

46. Guzman D, Ann Y, Bruera E, Wu J, Williams JL, Najera JM, et al. Enhancing palliative care patient access to psychological counseling through outreach telehealth services. Psycho-Oncology. 2020;29(1):7.

47. Badu E, O’Brien AP, Mitchell R. An integrative review on methodological considerations in mental health research – design, sampling, data collection procedure and quality assurance. Archives of public health = Archives belges de santé publique. 2019;77(1):37-15.

48. Iobst SE, Best N, Smith DC, Phillips MAK, Wilson C, Trego L. Promoting Military Women’s Health Through Research Design. Military medicine. 2023;188(3-4):71-6.

49. Carolan CM, Forbat L, Smith A. Developing the DESCARTE Model: The Design of Case Study Research in Health Care. Qualitative health research. 2016;26(5):626-39.

50. Følstad A, Araujo T, Papadopoulos S, Law L-C, Luger E, Goodwin M, et al. Chatbot research and design: 7th International Workshop, CONVERSATIONS 2023, Oslo, Norway, November 22-23, 2023, Revised selected papers. Cham, Oslo Norway: Springer; 2024.

51. Hendrix SL. Mobile Apps for Relationship Improvement: A Scoping Review. E-Health Telecommunication Systems and Networks 2025;14:10.

52. Blair L, Blair L. Ring of fire: An Indonesian odyssey. Singapore: Editions Didier Millet; 2010.

The original article [1] has been updated.

## References

[1] Purba CIH, Anggraeni R, Pahria T. Exploration of patients and multidisciplinary point of view about the self-care needs of patients with a stoma: a qualitative study. BMC Cancer. 2026;26:647. 10.1186/s12885-026-15920-8. 41963854 PMC13188443

